# Calcified mass attached to mitral valve chordae tendineae in a dog with hyperadrenocorticism: A case report

**DOI:** 10.1186/s12917-025-05172-2

**Published:** 2025-12-05

**Authors:** Tomoki Wada, Ryosuke Takemura, Hideki Yotsuida, Tomoya Okano, Takuya Mori

**Affiliations:** 1 Japan Animal Cardiovascular Care Team, 3-15-27 Hishie, Higashiosaka-shi, Osaka, 578-0984 Japan; 2 Kinki Animal Medical Center, 3-15-27, Hishie, Higashiosaka-shi, Osaka, 578- 0948 Japan; 3 National Cerebral and Cardiovascular Center, Suita-shi, Osaka, 564-8565 Japan; 4 FUJIFILM VET Systems Co., Ltd, 1-49-18 Nishikokubunji, Kokubunji-shi, Tokyo, 185-0013 Japan

**Keywords:** Calcification, Mitral valvuloplasty, Cushing syndrome, Myxomatous mitral valve disease

## Abstract

**Background:**

Dystrophic calcification affecting cardiac structures secondary to hyperadrenocorticism (HAC) is rare in dogs. This case report describes the clinical, imaging, surgical, and pathological findings of a 9-year-old spayed female Maltese dog with severe mitral regurgitation and HAC-related systemic calcification presenting as an intracardiac calcified mass.

**Case presentation:**

Severe mitral valve thickening and a mobile hyperechoic mass attached to the chordae tendineae were detected on echocardiography. Mitral valvuloplasty was performed, and the calcified mass was excised. Histopathology confirmed a non-neoplastic calcified lesion. Computed tomography revealed extensive calcification in various tissues. The dog developed postoperative aspiration pneumonia and died on postoperative day 2.

**Conclusions:**

This case highlights the potential for intracardiac dystrophic calcification in dogs with HAC and severe mitral valve disease.

**Supplementary Information:**

The online version contains supplementary material available at 10.1186/s12917-025-05172-2.

## Background

Calcification in dogs can be classified as metastatic calcification associated with hypercalcemia [1–3], dystrophic calcification occurring in necrotic or damaged tissues despite normal serum calcium levels, iatrogenic calcification due to medication, and idiopathic calcification of unknown etiology [4, 5]. Among these, dystrophic calcification occasionally occurs secondary to hyperadrenocorticism (HAC), in which prolonged hypercortisolism induces mineral deposition in multiple organs, including the skin and lungs, causing functional impairment and inflammation [4, 5]. Although dystrophic calcification has been documented in various tissues such as the skin, trachea, kidneys, liver, gastric mucosa, skeletal muscle, cornea, and the bifurcation of the abdominal aorta, explicit reports describing cardiac calcification in dogs are lacking [4, 6].

In humans, cardiac calcification is often associated with aging, valvular disease, healed myocardial infarction, ventricular aneurysm, postoperative scarring following cardiac surgery, and resolved infective endocarditis, frequently involving the cardiac skeleton or valve annulus [7, 8]. Rarely, mitral annulus calcification manifests as a mass-like lesion, necessitating differentiation from cardiac tumors or thrombi [9]. Additionally, in patients on dialysis or with valvular disease, non-neoplastic calcified lesions, termed calcified amorphous tumors (CAT), can form intracardially, displaying mobility and mimicking cardiac tumors [10]. However, to our knowledge, no reports have described similar nonneoplastic calcified cardiac masses in dogs.

In this report, we describe a dog with severe mitral regurgitation (MR) and systemic dystrophic calcification associated with HAC. During mitral valvuloplasty (MVP), a mobile calcified mass attached to the chordae tendineae was discovered and pathologically identified as a non-neoplastic calcified lesion. We present the clinical, surgical, and histopathological details of this unique case.

## Case presentation

A 9-year-old spayed female Maltese weighing 3.14 kg presented with a body condition score of 3/9 and severe muscle wasting. The dog had a medical history of pulmonary edema due to mitral valve regurgitation and was referred for MVP. Medications prescribed by the referring veterinarian included alacepril (2.4 mg/kg every 12 h), pimobendan (0.5 mg/kg every 8 h), spironolactone (2.5 mg/kg every 12 h), and torasemide (0.27 mg/kg every 12 h). The dog had no history of steroid use; its water intake exceeded 400 mL/day.

On initial examination, the heart rate was 126 beats per minute with a regular rhythm. Femoral pulses and mucous membrane color were normal. Abdominal distention, skin thinning, and focal areas of firm skin hardening were observed. Auscultation revealed a grade 4/6 systolic murmur at the left apex. Thoracic radiography revealed cardiac enlargement (vertebral heart score = 12.5; [reference interval [RI]: 8.7–10.7 [11]) without pulmonary edema. Two-dimensional echocardiography was performed using a Vivid E95 ultrasound system (GE Healthcare, Tokyo, Japan), confirming severe MR, characterized by prolapse and thickening of the anterior mitral leaflet, left atrial enlargement (left atrial-to-aortic diameter ratio, 2.37 [RI: <1.6] [12]), normalized left ventricular internal dimension in diastole of 2.15 [RI: 1.2–1.64] [13], and left ventricular end-diastolic diameter of 30.1 mm (RI: 15–22 mm) [14]. Based on these findings, the dog was diagnosed as Stage C, and MVP was planned according to the current guidelines [15]. Suspecting HAC, adrenocorticotropic hormone (ACTH) stimulation testing was performed by a primary veterinarian. An ACTH stimulation test confirmed hyperadrenocorticism (pre-ACTH cortisol: 22.1 µg/dL, post-ACTH cortisol: >50 µg/dL), and trilostane (Trilostab; Fujita Pharmaceutical, Tokyo, Japan) (0.4 mg/kg every 12 h) was initiated. On day 38, surgery was postponed because the dog experienced vomiting, diarrhea, and respiratory distress. An ACTH stimulation test performed at the primary veterinarian revealed pre- and post-ACTH cortisol concentrations of 13.4 and 17.0 µg/dL, respectively. Serum biochemistry (IDEXX Laboratories (Westbrook, ME, USA) revealed elevated blood urea nitrogen level (29 mg/dL [10.4 mmol/L]; RI: 7–27 mg/dL [2.5–9.6 mmol/L]), decreased creatinine level (0.4 mg/dL [35.4 µmol/L]; RI: 0.5–1.8 mg/dL [44.2–159.1 µmol/L]), and normal calcium level (9.1 mg/dL [2.27 mmol/L]; RI: 7.9–12.0 mg/dL [1.98–3.00 mmol/L]) and phosphorus level (5.2 mg/dL [1.68 mmol/L]; RI: 2.5–6.8 mg/dL [0.81–2.20 mmol/L]). Thoracic radiography revealed an increased opacity in the right middle lung lobe, consistent with aspiration pneumonia. The dog was treated with antibiotics, antiemetics, and antidiarrheal medications and was discharged on day 46 with continued trilostane therapy.

**Fig. 1:**
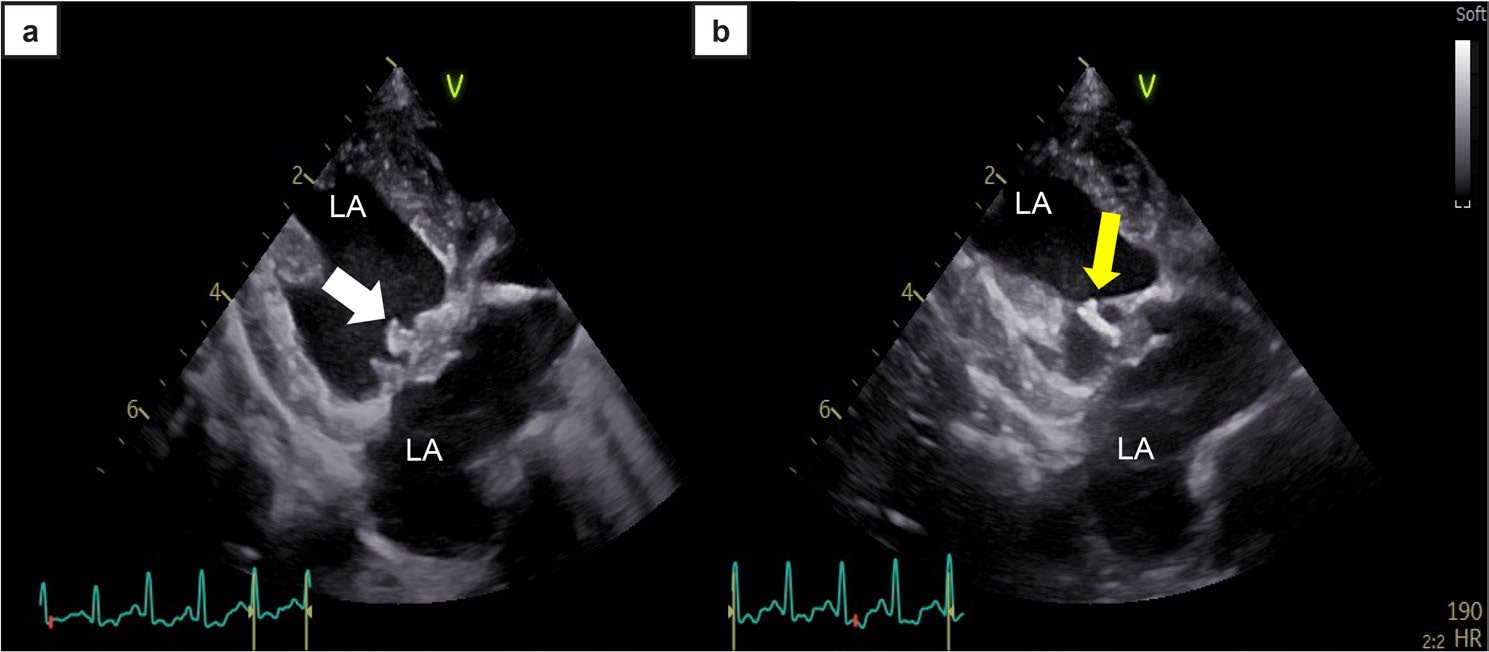
Two-dimensional echocardiographic images obtained on the day of surgery **a**, **b**. The right parasternal long-axis view demonstrates a hyperechoic structure beneath the mitral valve (white arrow). The mitral valve chordae tendineae also exhibit increased echogenicity (yellow arrow). LA: left atrium, LV: left ventricle

By day 107, the dog’s body weight had decreased to 2.4 kg, with a body condition score of 1/9, and persistent severe muscle wasting. The skin became diffusely rigid and plate-like, and a firm white structure was observed on the tongue. Corneal ulceration with superficial opacity suggestive of calcific band keratopathy was observed. Water intake ranged from 300 to 400 mL/d. Serum biochemistry (IDEXX Laboratories) revealed elevated blood urea nitrogen level (52 mg/dL [18.6 mmol/L]) and normal creatinine (0.5 mg/dL [44.2 µmol/L]), calcium (11.3 mg/dL [2.82 mmol/L]), and phosphorus levels (6.8 mg/dL [2.20 mmol/L]). Arterial blood gas analysis revealed an ionized calcium concentration of 1.53 mmol/L (RI: 1.24–1.56 mmol/L). Owing to imminent surgery, only the baseline (pre-ACTH) cortisol concentration was measured, revealing 19.5 µg/dL. Echocardiography revealed a mobile structure attached to the mitral valve, suspected to be ruptured chordae (Fig. 1a, Additional files 1 and 2), along with increased echogenicity of the left ventricular myocardium and chordae tendineae (Fig. 1b).

**Fig. 2:**
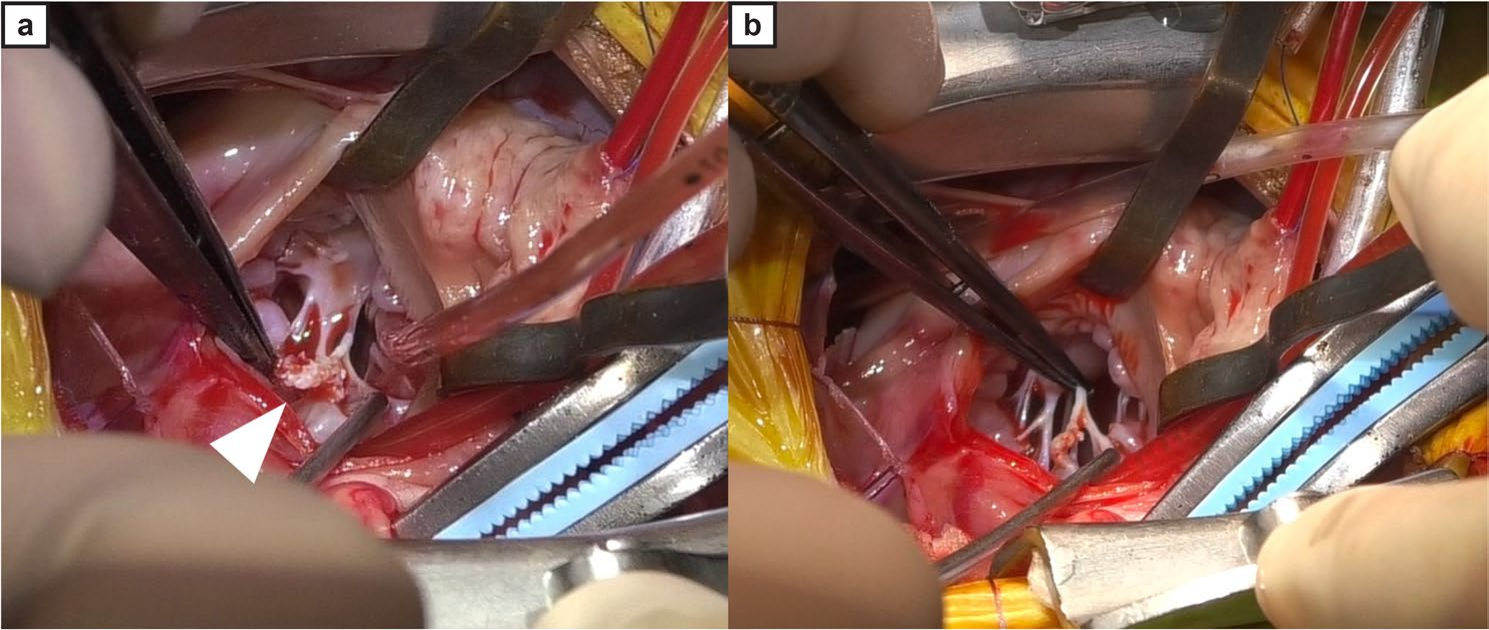
Intraoperative view of the mitral valve and chordae tendineae after mass removal. Intraoperative images show an irregular, firm mass attached to the chordae tendineae of the anterior mitral valve leaflet (**a**, white arrowhead). After the mass is removed, the exposed chordae exhibit white, irregular, and uneven margins (**b**)

On day 108, an MVP involving chordal reconstruction and annuloplasty was performed under cardiopulmonary bypass. The patient was premedicated with fentanyl (5 µg/kg intravenously). Anesthesia by propofol was induced, and the dog was intubated with a cuffed endotracheal tube. Anesthesia was maintained by inhalation of isoflurane in 100% oxygen and constant rate infusions of fentanyl (1 µg/kg/h), remifentanil (20 µg/kg/h) and propofol (0.2–0.4 mg/kg/min). Muscle relaxation was achieved by intermittent administration of rocuronium bromide (0.5 mg/kg intravenously). Cannulation was achieved via the left jugular vein (venous return) and left carotid artery (arterial perfusion). After a thoracotomy through the left fifth intercostal space, cardioplegic arrest was achieved by placing a root cannula on the aortic root. Inspection of the mitral valve through a left atriotomy revealed a firm mass attached to the ruptured chordae tendineae (Fig. 2). The mass easily detached during manipulation (Additional file 3). The remaining chordae were white and sclerotic, with additional firm white areas observed in the rough zone of the anterior mitral leaflet, fibrous trigone, and posterior papillary muscle. The chordae tendineae in regions 1–2 and 2–3 of the anterior leaflet were excised because of calcification. Chordal reconstruction and annuloplasty were performed, followed by left atrial closure and thoracotomy. Postoperative laboratory tests revealed parathyroid hormone-related protein levels of 1.3 pmol/L (RI: ≤2.0 pmol/L) and intact parathyroid hormone levels of 6.1 pg/mL (RI: 1.4–16.2 pg/mL).

**Fig. 3:**
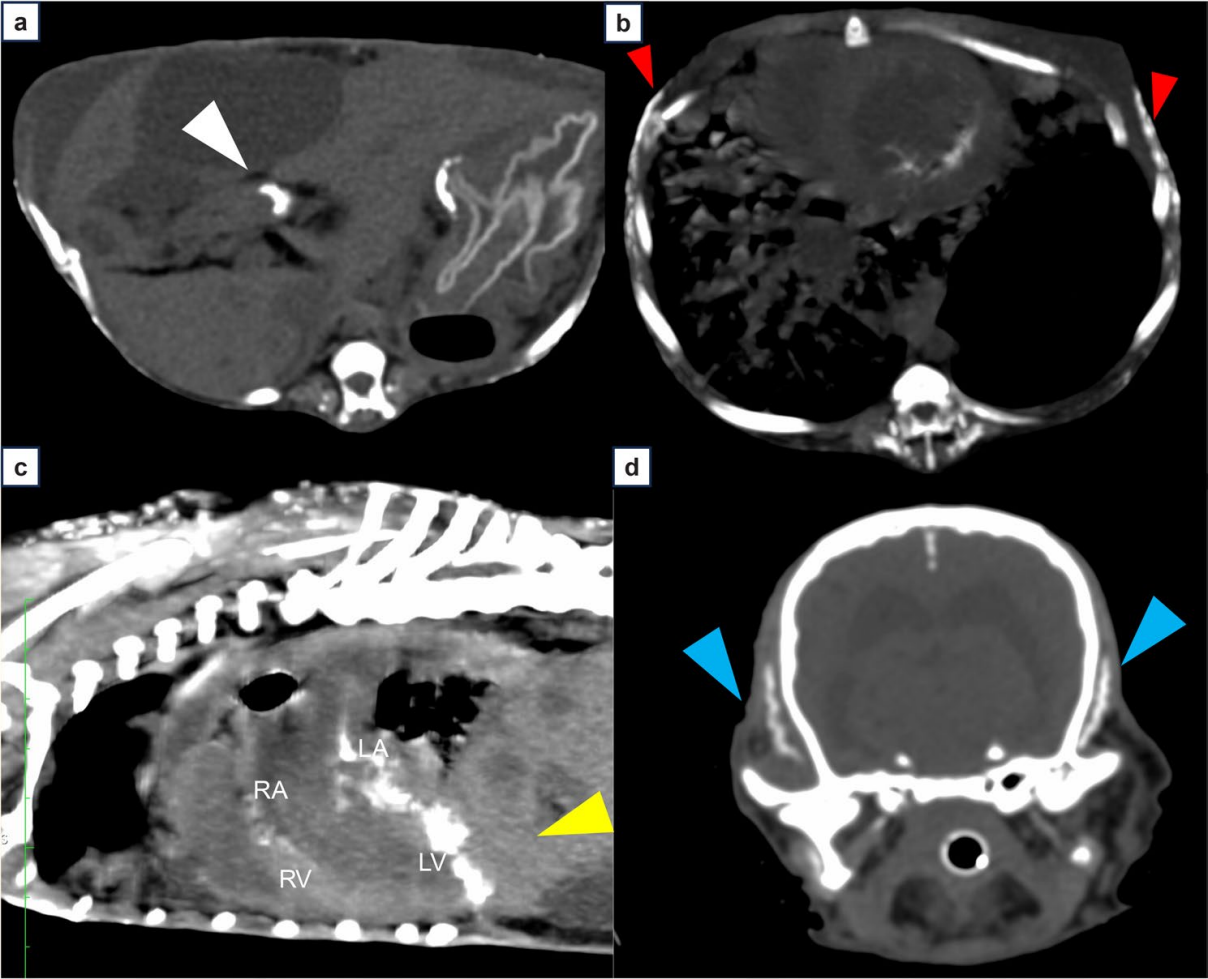
Computed tomography images showing systemic calcification in multiple tissues. The images show systemic calcification in several soft tissues, including gastric mucosa (**a**, white arrow), subcutaneous tissue (**b**, red arrowheads), left ventricle (**c**, yellow arrow), and temporal muscle (**d**, blue arrowhead)

The day after surgery, the respiratory condition of the dog worsened following vomiting, and an anesthetized computed tomography scan suggested aspiration pneumonia. Computed tomography imaging (Revolution CT scanner; GE Healthcare, Tokyo, Japan) revealed extensive calcification in the subcutaneous tissue, skeletal muscle, gastric mucosa, tongue, and left ventricle (Fig. 3, Additional file 4), and pituitary enlargement (5 mm). Despite intensive management, the respiratory function did not improve, and the dog died on day 110 due to systemic inflammatory response syndrome and hypotension. A post-mortem examination was performed.

**Fig. 4:**
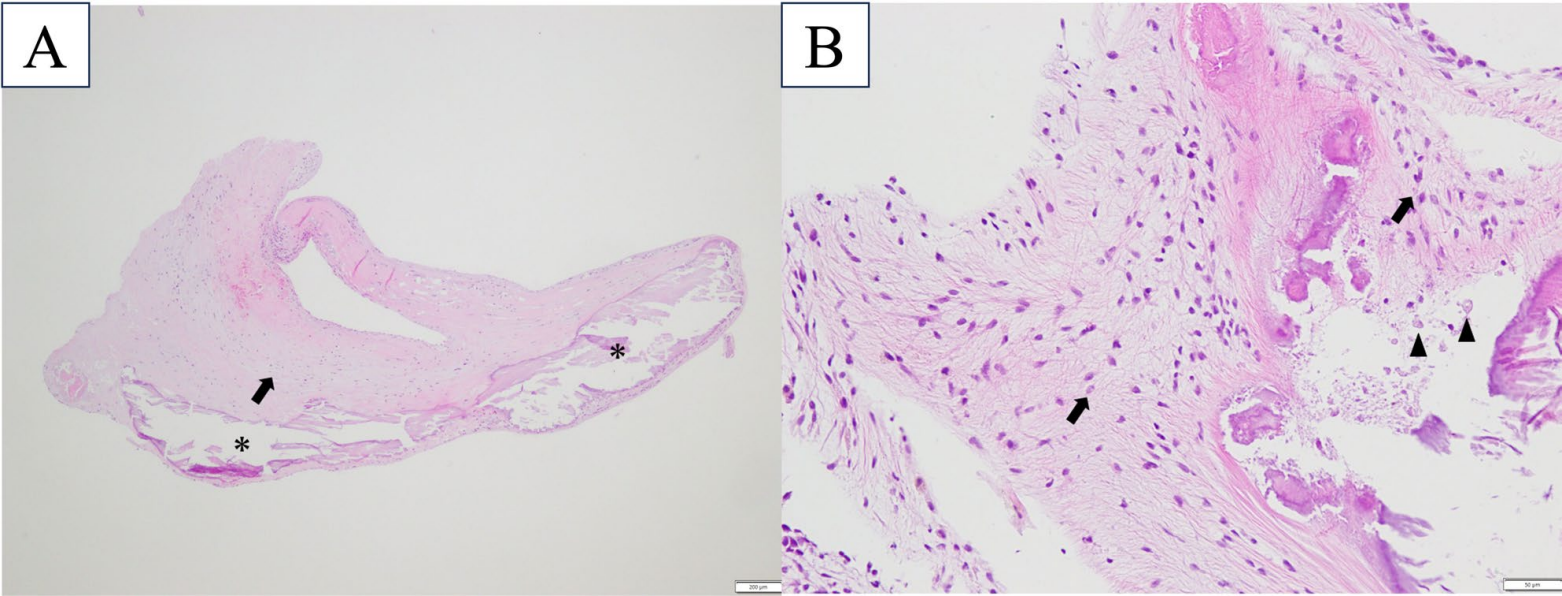
Histopathological findings of the intracardiac mass surgically excised. (a) Low-power photomicrograph showing aggregates of calcified material (asterisks) surrounded by mildly edematous stroma with fibroblast proliferation (arrows). Scattered macrophage infiltration (arrowheads) is observed in the interstitium. No evidence of cellular atypia or infectious organisms is noted (hematoxylin and eosin staining, magnification 40×, scale bar = 200 μm). (b) High-power view illustrating laminated calcified deposits (asterisks) embedded within fibrous tissue, accompanied by adjacent proliferative fibroblasts (arrows) and infiltrating macrophages (arrowheads). No neoplastic changes are identified (hematoxylin and eosin staining, magnification 200×, scale bar = 20 μm) Additional files Additional files 1 and 2. Echocardiographic visualization of abnormal structures. Mobile hyperechoic structures attached to the mitral valve and chordae tendineae exhibited oscillatory movements synchronized with the cardiac cycle. (mp4) Additional file 3**.** Intraoperative observation of the mitral valve after cardiac arrest. The irregular mass attached to the chordae tendineae was easily detached with gentle manipulation. The remaining chordae tendineae were white and sclerotic. (mov) Additional file 4. Three-dimensional reconstructed CT images demonstrate extensive calcification on the dog’s surface. Prominent calcification was evident at the costochondral junctions and around the scapula. (mov)

Histological evaluation of the chordae tendineae obtained during surgery revealed mild to moderate calcification and fibroblast proliferation. The calcified mass, detached from the chordae, was predominantly composed of calcified clusters, fibroblasts, and mild macrophage infiltration (Fig. 4a, b). Histopathological examination revealed coronary artery calcification, diffuse myocardial necrosis, fibrosis, and severe irregular thickening of the mitral valve leaflet with fibroblast proliferation, myxomatous edema, and scattered degenerate neutrophils. Gastric mucosal calcification, diffuse adrenal hyperplasia, and a small nodule in the right adrenal medulla suspected to be a cortical adenoma were also observed. Extensive aspiration pneumonia, hepatic steatosis with nodular hyperplasia, and mild acute pancreatitis were observed. No significant lesions were observed in the kidneys, trachea, urinary bladder, or colon. Bacterial culture of the sputum obtained postmortem via an endotracheal tube yielded *Enterococcus faecium*, suggesting aspiration pneumonia and sepsis as the causes of death.

##  Discussion and conclusions

Dystrophic calcification occasionally occurs in dogs with HAC, resulting in calcium deposition across various tissues. Calcinosis cutis is the most frequently reported manifestation, occurring in 1.7–8% of early diagnosed cases and up to 40% of advanced cases [16]. Additionally, calcification can affect pulmonary structures (tracheal and bronchial walls), kidneys, gastric mucosa, liver, skeletal muscle, cornea, and blood vessel walls [5, 6]. The mechanism by which cortisol induces dystrophic calcification is thought to involve not only its gluconeogenic and proteolytic effects but also local ischemia caused by adipocyte hypertrophy associated with hypercortisolism [17]. Necrosis or degeneration of these tissues leads to the release of enzymes, resulting in calcium accumulation within mitochondria through interaction with the electron transport system. During this process, an equivalent amount of phosphate is incorporated, accompanied by the degradation of organic phosphate compounds and changes in pH. Consequently, calcium phosphate is deposited as granular matrix inclusions, leading to degenerative (dystrophic) calcification [18]. In the present case, calcification was observed in the skin, gastric mucosa, heart, tongue, and cornea despite normal serum calcium and phosphorus concentrations, suggesting dystrophic calcification due to hyperadrenocorticism. However, calcification previously reported in the lungs, kidneys, and liver was not evident here, highlighting the variability in calcification distribution among cases. Moreover, diagnosis may be challenging depending on the calcification site, especially within cardiac and vascular tissues.

Cardiac calcification is rare in dogs. Although incidental calcification of the aortic root on thoracic radiographs has been reported in older dogs, these findings generally reflect aging or chronic degeneration without clinical significance [19]. Intracardiac calcification, particularly involving the chordae tendineae or cardiac valves, has rarely been documented [20]. Increased valvular echogenicity suggestive of calcification has been observed in chronic mitral valve disease and infective endocarditis [21, 22], but severe intracardiac calcification remains exceptional. In the present case, significant calcification was identified within the left ventricle, which formed a hardened, calcified mass attached to the mitral valve chordae tendineae, likely associated with underlying hyperadrenocorticism. This is the first report of an intracardiac calcified mass in a dog with HAC. Preoperatively, the mass demonstrated mobility, and intraoperative manipulation easily dislodged it, raising concerns about potential embolization if left untreated. Previous reports have described myocardial infarction secondary to coronary arteriosclerosis in dogs with iatrogenic Cushing’s syndrome [4]. Considering the coronary artery calcification observed in this case, veterinary clinicians should be aware of the increased cardiovascular risk associated with hyperadrenocorticism.

In this case, the presence of severe cardiac calcification could not be diagnosed preoperatively. Although radiography is known to have diagnostic accuracy comparable to that of CT for cutaneous calcinosis [23], CT is superior for detecting cardiac calcification [24]. In this dog, neither thoracic radiography nor echocardiography raised suspicion of calcification before surgery; the finding was identified only on postoperative CT. In humans, calcification of the mitral apparatus is recognized as a risk factor that increases operative mortality and the incidence of postoperative complications such as acute kidney injury and respiratory failure [25]. It also influences the choice of surgical technique: in cases with severe calcification of the mitral annulus, mitral valve replacement is often preferred over mitral valve repair [26]. Moreover, when the valvular leaflets themselves are heavily calcified and fragile, their plasticity is lost, making repair infeasible and replacement necessary [26]. Although mitral valve repair in dogs with calcification of the mitral apparatus has not been investigated, such calcification should be considered a potential surgical risk factor.

The intracardiac calcified lesions observed in this study differed histologically from the calcified CATs described in human medicine. CATs are characterized by fibrin-like amorphous matrices with calcium deposits [27]. Conversely, the lesion in our case primarily comprised clustered calcifications, fibroblast proliferation, and mild macrophage infiltration without fibrin-like deposition or amorphous eosinophilic material, indicating a distinct histological entity. The differential diagnosis of intracardiac calcified lesions should include tumors, thrombi, and infective endocarditis vegetations [27]. Preoperatively, the mobile lesion was indistinguishable from tumors or thrombi solely on echocardiography, highlighting the critical role of surgical excision and histopathological analysis in a definitive diagnosis.

A notable aspect of this case was the coexistence of severe mitral valve regurgitation and Cushing’s syndrome, which potentially contributed to the pronounced cardiac calcification. In humans, calcification predominantly affects the aortic and mitral valves owing to endothelial damage and inflammation induced by high pressure and flow, triggering active calcification processes [28]. Multiple factors, including aging, mechanical stress, local inflammation, and hemodynamic disturbances, contribute to valvular calcification [29–32]. Mechanical strain activates the valvular interstitial cells and promotes excessive extracellular matrix production, fibrosis, and calcification [30–32]. Both human and canine studies have indicated that increased pressure load and mechanical stress can induce osteoblast-like differentiation of valvular interstitial cells, facilitating calcium deposition in the extracellular matrix [20]. In addition, local inflammation plays a significant role, as inflammatory cell infiltration has been observed in calcified human mitral annuli [33]. Endothelial cell injury caused by inflammation or shear stress can induce endothelial-to-mesenchymal transition, further activating valvular interstitial cells and initiating fibrotic responses [20, 30, 31]. Under chronic stimulation, these cells differentiate into osteoblast-like phenotypes, express osteogenic markers such as RUNX2 and bone alkaline phosphatase, and promote hydroxyapatite deposition [20, 30]. In severe cases, calcification may extend to the left ventricle, papillary muscles, chordae, left ventricular outflow tract, and rarely to the aorto-mitral curtain [33]. Fox reported characteristic histological changes in dogs with myxomatous mitral valve disease, including glycosaminoglycan and proteoglycan accumulation within the spongiosa layer, disruption and attenuation of the fibrosa layer, and phenotypic transition of valvular interstitial cells from a fibroblast-like to a myofibroblast-like form [34]. In advanced conditions, nodular thickening and deformation of the mitral valve leaflets occur, along with proximal thickening of the chordae tendineae and rupture of the internal collagen bundles. Such degenerative lesions can serve as substrates for dystrophic calcification [20, 34]. Accordingly, the marked intracardiac calcification observed in this case may have been exacerbated by severe mitral regurgitation with chordal rupture, structural degeneration of the mitral valve leaflets and chordae tendineae, and inflammatory cell infiltration. In other words, chronic valve tissue degeneration characterized by myxomatous changes and fibrosis may have facilitated dystrophic calcification within the mitral valve apparatus and left ventricular structures, in addition to the systemic dystrophic calcification associated with HAC.

In conclusion, this case illustrates a severe systemic calcification secondary to advanced HAC complicated by mitral valve disease. Although dystrophic calcification associated with HAC is generally recognized as calcinosis cutis, advanced disease progression may result in widespread systemic calcification, as was observed in this case. This multifactorial condition, including systemic metabolic disturbances due to HAC, combined with local mechanical stress from severe valvular disease, likely contributes to the formation of pseudotumor-like intracardiac calcified lesions. The emergence of intracardiac calcification may adversely affect the prognosis, highlighting the critical importance of therapeutic interventions in dogs with HAC. This report provides valuable insights into severe systemic complications resulting from poorly controlled HAC, emphasizing the need to accumulate and investigate similar cases in the future.

## Supplementary Information

Supplementary Material 1.

Supplementary Material 2.

Supplementary Material 3.

Supplementary Material 4.

## Data Availability

All datasets used and/or analyzed during the current study are available from the corresponding author on reasonable request.

## Abbreviations

ACTH: adrenocorticotropic hormone
CAT: calcified amorphous tumors
HAC: hyperadrenocorticism
MR: mitral regurgitation
MVP: mitral valvuloplasty
RI: reference interval
